# Modeling the burden of long COVID in California with quality adjusted life-years (QALYS)

**DOI:** 10.1038/s41598-024-73160-x

**Published:** 2024-09-30

**Authors:** Sophie Zhu, Kalyani McCullough, Jake M. Pry, Seema Jain, Lauren A. White, Tomás M. León

**Affiliations:** 1 Division of Communicable Disease Control, California Department of Public Health, Richmond, USA; 2 Department of Pathology, Microbiology, and Immunology, University of California, Davis, USA; 3 Department of Public Health Sciences, University of California, Davis, USA

**Keywords:** Epidemiology, Computational models

## Abstract

Individuals infected with SARS-CoV-2 may develop post-acute sequelae of COVID-19 (“long COVID”) even after asymptomatic or mild acute illness. Including time varying COVID symptom severity can provide more informative burden estimates for public health response. Using a compartmental model driven by confirmed cases, this study estimated long COVID burden by age group (0–4, 5–17, 18–49, 50–64, 65+) in California as measured by the cumulative and severity-specific proportion of quality-adjusted life years (QALYs) lost. Long COVID symptoms were grouped into severe, moderate, and mild categories based on estimates from the Global Burden of Disease study, and symptoms were assumed to decrease in severity in the model before full recovery. All 10,945,079 confirmed COVID-19 cases reported to the California Department of Public Health between March 1, 2020, and December 31, 2022, were included in the analysis. Most estimated long COVID-specific QALYs [59,514 (range: 10,372–180,257)] lost in California were concentrated in adults 18–49 (31,592; 53.1%). Relative to other age groups, older adults (65+) lost proportionally more QALYs from severe long COVID (1,366/6,984; 20%). Due to changing case ascertainment over time, this analysis might underestimate the actual total burden. In global sensitivity analysis, estimates of QALYs lost were most sensitive to the proportion of individuals that developed long COVID and proportion of cases with each initial level of long COVID symptom severity (mild/moderate/severe). Models like this analysis can help translate observable metrics such as cases and hospitalizations into quantitative estimates of long COVID burden that are currently difficult to directly measure. Unlike the observed relationship between age and incident severe outcomes for COVID-19, this study points to the potential cumulative impact of mild long COVID symptoms in younger individuals.

**Supplementary Information:**

The online version contains supplementary material available at 10.1038/s41598-024-73160-x.

## Introduction

Post-acute sequelae of coronavirus disease 2019 (COVID-19), also known as “long COVID,” refers to lingering or emerging symptoms following severe acute respiratory syndrome coronavirus 2 (SARS-CoV-2) infection. Long COVID burden is difficult to measure due to the lack of a consensus case definition; the Centers for Disease Control and Prevention (CDC) defines long COVID as starting at least four weeks after infection^1^, while the World Health Organization defines long COVID as illness three months after COVID-19 infection that lasts for at least two months^2^. Many long COVID studies have been descriptive or clinical in nature, answering questions related to etiology, risk factors, and disease progression over time^3–5^. Accurate estimates of long COVID prevalence are needed to inform decisions related to unemployment benefits, proportion of people experiencing temporary and permanent disability, and equitable distribution of treatments and vaccines. According to a recent meta-analysis, roughly 31% of previously infected SARS-CoV-2 patients in North America experience long COVID at three months post-infection^6^. Because representative population prevalence estimates have been complicated by highly heterogeneous clinical and subclinical experiences related to symptom duration and severity, it is necessary to further refine estimates using models to understand the current burden of long COVID on patients and healthcare systems^7^.

One way that long COVID burden has been measured is through quality adjusted life-years (QALYs), a standardized value that measures the quantity and quality of years of life lost to disease and disability^8^, which can account for differences in symptom severity. Other studies have estimated disability weight (quality of life score; QoL) for long COVID based on similar post-illness conditions such as chronic fatigue syndrome^9^. Estimates for the proportion of people who experience long COVID vary depending on the population studied, definition of long COVID used, and time point^6^. Age, vaccination status, and dominant variant are also factors that are associated with major differences in infection, hospitalization, and mortality patterns, which alter the distribution of long COVID^10^. Mathematical modeling can complement existing studies by providing estimates of current burden that reflect parameter uncertainty^11^, exploring assumptions about symptom progression over time, supporting prioritization of parameter estimates for future modeling efforts, and contributing to scenario analyses for consideration by healthcare providers and policymakers^12^.

California is home to 12% (39.2 million) of the US population and is characterized by high demographic and socio-economic diversity. This heterogeneity is reflected in differences in vaccine uptake, use of non-pharmaceutical interventions, and access to testing, treatments, and health services across the state, which has resulted in disparities in SARS-CoV-2 infection, hospitalization, and mortality, as well as long COVID outcomes^13–16^. Several ongoing observational studies seek to measure risk factors and proportion of people experiencing long COVID^17,18^; however, there is no current estimate of the statewide long COVID burden, which would be useful to inform local allocation of healthcare and social resources.

The objective of this study was to estimate the long COVID burden in California based on confirmed COVID-19 cases that occurred between March 1, 2020, and December 31, 2022, using a mathematical modeling approach to estimate the impact of long COVID burden disaggregated by symptom severity in California. Age-specific infection, hospitalization, and mortality rates were included to capture changing burdens more accurately over time. Model parameters were evaluated using Latin Hypercube Sampling (LHS) and partial rank correlation coefficients in a global sensitivity analysis to identify the most influential parameters for the outcomes of interest.

## Methods

### Model structure

**Fig. 1:**
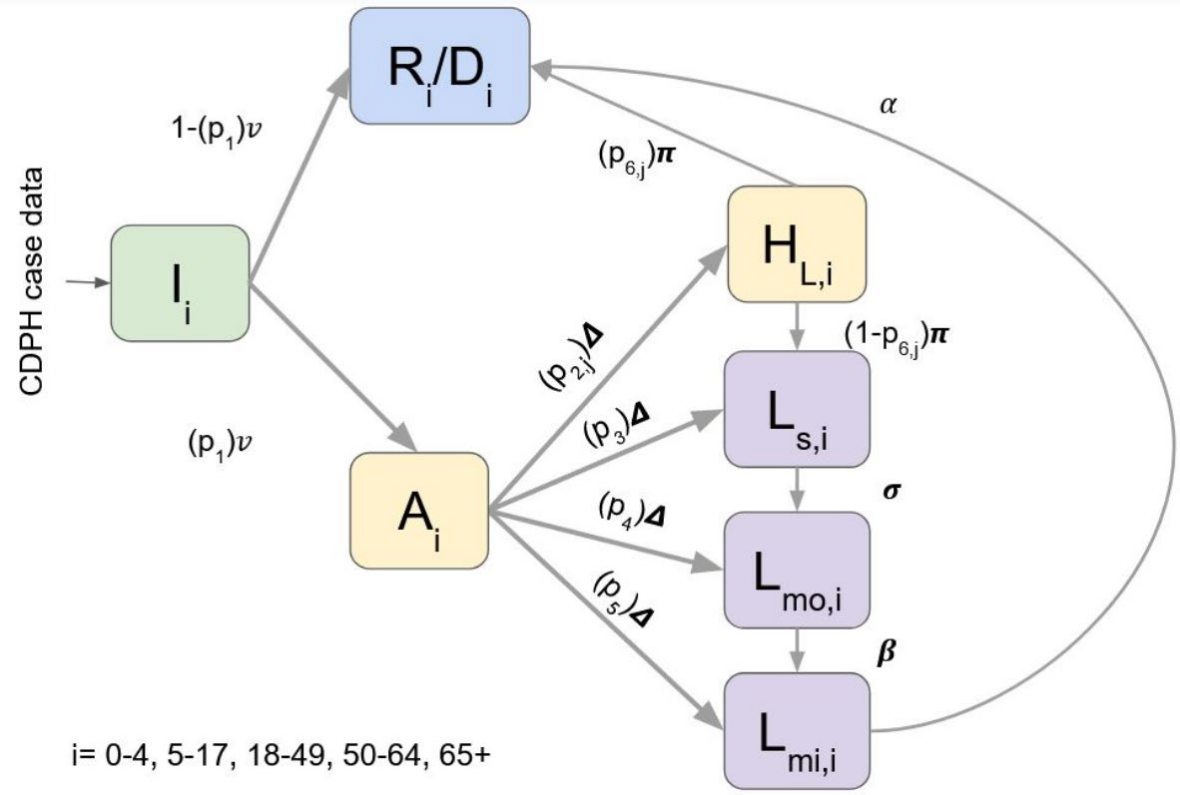
Structure of compartmental model used to track long COVID burden in California. *I* is all COVID-19 cases. *R/D* represents infected persons who recover completely or die and do not develop long COVID. *A* is the cohort of COVID-19 cases who will develop long COVID. *H*_*L*_ represents hospitalized cases that will progress to severe long COVID or recovery/death (*R/D)*. *L*_*s*_, *L*_*mo*_, and *L*_*mi*_ are individuals that experience severe, moderate, and mild long COVID symptoms, respectively. Each compartment was divided across five age categories ‘i’ (0–4, 5–17, 18–49, 50–65, 65+). Parameters *p*_2_ and *p*_6_ also capture hospitalization and mortality rates across three time periods (ancestral and Alpha, Delta, and Omicron) denoted by the subscript ‘j’.

We developed a deterministic age-structured compartmental model of long COVID progression over time (Fig. 1). Large scale surveys of long COVID patients show that, in general, individuals experience a decrease in symptom severity over time^7^. Therefore, we assumed that incident COVID-19 severity was correlated with initial symptom severity of long COVID and long COVID patients experienced reduced symptom severity before recovery. COVID-19 cases (*I*) could either recover completely or die (*R/D*) without experiencing long COVID (including some persons who are hospitalized) or be part of the cohort of cases who will develop long COVID (*A*). Of this cohort (*A*), cases can be hospitalized (*H*_*L*_*)* before progressing to recovery/death (*R*/*D*) or severe long COVID, or progress directly to long COVID at one of three long COVID severity levels (Fig. 1): severe (*L*_*sev*_), moderate (*L*_*mod*_), or mild (*L*_*mild*_), which correspond to the Global Burden of Disease Study’s disability weighting (see *Model parameterization* below)^9^. The model can be represented by the following equations:

$$\begin{aligned} & dI/dt = - ({{1}} - p_{1} )\;{{\Delta }}_{i} I\left( t \right)~ - p_{1} \nu _{i} I\left( t \right) \\ & dR/D/dt = ({{1}} - p_{1} )\;\nu _{i} I\left( t \right) + \alpha _{i} L_{{mild}} \left( t \right) + ~p_{6} \pi _{i} H_{L} \left( t \right) \\ & dA/dt = ~p_{1} \nu _{i} I\left( t \right) - p_{2} {{\Delta }}_{i} A\left( t \right) - p_{3} {{\Delta }}_{i} A\left( t \right) - p_{4} {{\Delta }}_{i} A\left( t \right) - p_{5} {{\Delta }}_{i} A\left( t \right) \\ & dH_{L} /dt = ~p_{2} {{\Delta }}_{i} A\left( t \right) - p_{6} \pi _{i} H_{L} \left( t \right) - \left( {1 - p_{6} } \right)\pi _{i} H_{L} \left( t \right) \\ & dL_{{sev}} /dt = p_{3} {{\Delta }}_{i} A\left( t \right) + \left( {1 - p_{6} } \right)\pi _{i} H_{L} \left( t \right) - \sigma _{i} L_{{sev}} \left( t \right) \\ & dL_{{mod}} /dt = ~p_{4} \Delta _{i} A\left( t \right) + \sigma _{i} L_{{sev}} \left( t \right) - \beta _{i} L_{{mod}} \left( t \right) \\ & dL_{{mild}} /dt = ~p_{5} \Delta _{i} A\left( t \right) + \beta _{i} L_{{mod}} \left( t \right) - \alpha _{i} L_{{mild}} \left( t \right) \\ \end{aligned}$$

Parameters are defined in Table 1 and the subscript *i* denotes five age categories (0–4, 5–17, 18–49, 50–65, 65 + years old). Parameters *p*_2_ (percentage of individuals hospitalized due to acute infection) and *p*_6_ (percentage of individuals dying of SARS-CoV-2 infection) are age and COVID variant period stratified, denoted by subscripts i and j respectively.

**Table 1:** Estimates and sources for parameters used in long COVID compartmental model in California.

Parameter	Description	Estimate	Distribution	Citation
*ν*	Rate at which an individual progresses from positive test result to recovery or long COVID onset	1/14 days^−1^	Unif~(1/28^a^, 1/6.87)	^19^
$\varDelta$	Rate at which an individual progresses from end of acute infection to beginning of long COVID symptoms	1/14 days^−1^	Unif~(1/76, 1/14)	^2^
π^b^	Rate at which hospitalized individuals progress to death or develop severe long COVID symptoms	1/5 days^−1^	Unif~(1/6, ¼)	CDPH
$\sigma$ ^c, d^	Rate of symptom improvement from severe to moderate long COVID	1/40.66 days^−1^	Unif~(1/81.33, 1/40.66)	^9^
β^c, d^	Rate of symptom improvement from moderate to mild long COVID	1/81.33 days^−1^	Unif~(1/81.33, 1/40.66)	^9^
^d, e^	Rate of recovery for mild long COVID symptoms	1/92 days^−1^	Unif~(1/110, 1/80)	^9^
*p*_1_^f^	Proportion infected that develop long COVID	0.31	Triangle~(0.09, 0.53, 0.31)	^6^
*p*_2_^b^	Percentage of individuals infected that will develop long COVID who become hospitalized during acute infection	(Age: Alpha, Delta, Omicron)^g^ 0–4: 0.016, 0.015, 0.011 5–17: 0.007, 0.005, 0.002 18–49: 0.024, 0.021, 0.007 50–64: 0.078, 0.065, 0.019 65+: 0.222, 0.167, 0.083	Unif~(0.005, 0.016) Unif~(0.002, 0.007) Unif~(0.007, 0.024) Unif~(0.019, 0.078) Unif~(0.083, 0.222)	CDPH (eFigure 1)
*p*_3_	Proportion severe LC	0.005	Unif~(0.0025, 0.0075) *-50% and + 50% for lower/upper bounds	NIH data
*p*_4_	Proportion moderate LC	0.117	Unif~(0.0585, 0.175) *-50% and + 50% for lower/upper bounds	NIH data
*p*_5_	Proportion mild LC	0.878	Unif~(0.439, 0.99) *-50% lower bound and upper limit of 0.99	NIH data
*p*_6_^b^	Percentage of hospitalized individuals who die due to SARS-CoV-2 infection and related complications	(Age: Alpha, Delta, Omicron)^g^ 0–4: 0.006, 0.009, 0.005 5–17: 0.008, 0.009, 0.011 18–49: 0.085, 0.107, 0.051 50–64: 0.231, 0.237, 0.159 65+: 0.541, 0.390, 0.289	Unif~(0.006, 0.009) Unif~(0.008, 0.011) Unif~(0.051, 0.107) Unif~(0.159, 0.237) Unif~(0.289, 0.541)	CDPH
QoL_mild_	Disability weight of individuals with mild respiratory and cognitive problems (cough and shortness of breath after physical exertion, difficulty in concentrating and remembering recent events)	0.045 (0.005, 0.10)	Uncertainty Interval^h^ (0, 0.10), used Unif~(0, 0.10) in sensitivity analysis	^9^
QoL_mod_	Disability weight of individuals with moderate chronic respiratory and/or persistent fatigue with bodily pain or mood swings (cough and shortness of breath after light physical activity, always feel fatigue and may have depression)	0.225 (0.15–0.31)	UI^h^ (0.15–0.31), used Unif~(0.15–0.31) in sensitivity analysis	^9^
QoL_sev_	Disability weight of individuals with severe chronic respiratory problems and cognitive problems (cough and shortness of breath all the time, disorientation, memory problems, and confusion to where one needs help with some daily activities)	0.395 (0.20–0.59)	UI^h^ (0.20–0.59), used Unif~(0.20–0.59) in sensitivity analysis	^9^

^a^28 days set as upper bound based on CDC definition of long COVID, lower limit from Menni et al.

^b^Parameter based on confirmed cases reported to the California Department of Health only and may not be reflective of all observed and unobserved COVID cases/infections.

^c^Assumed people would spend less time in severe versus moderate (1:2).

^d^Mean Long COVID symptom cluster duration was 4.0 months (95% UI, 3.6–4.6 months) among non hospitalized individuals. Subtracted 28 days from mean symptom duration for LC severe, moderate, and mild to account for time with acute infection captured in and .

^e^Mild symptom recovery rate $\left({\upalpha}\right)$^9^ originally sampled from a normal distribution derived from Bayesian hierarchical modeling. A uniform distribution using the 95% CI was used to restrict LHS to sampling positive values only.

^f^Original distribution for *p*_1_ = Norm~(0.31, 0.11) obtained from a systematic review and meta-analysis for the proportion of individuals with long COVID. Because sampling from this distribution allowed values to be negative, we used a triangle distribution with mean 0.31 and 95% CI from the original distribution (minimum 0.09, and maximum of 0.53) to restrict LHS to positive values only.

^g^Alpha time period defined as 3/1/2020-5/31/2021, Delta 6/1/2021-12/31/2021, and Omicron 1/1/2022-12/31/2022.

^h^Uncertainty interval (UI) used because the parameter was obtained from a study that used Bayesian mixed-effects modeling.

Although asymptomatic individuals can develop long COVID^15,20^, we did not explicitly consider symptomatic and asymptomatic SARS-CoV-2 cases separately because asymptomatic cases are often combined with mild cases in published reports^21^.

### Quality-adjusted life years

This study only accounts for years of life lost due to disability, not death, and are calculated by applying a severity specific QoL score to the cumulative person-days that individuals spend in each long COVID severity level from time (*t*) = 0 to time *t = j* (end of study period) such that:

$$QALYs=\frac{Qo{L}_{sev}}{365}\sum_{t=0}^{j}{L}_{sev}\left(t\right)+\frac{Qo{L}_{mod}}{365}\sum_{t=0}^{j}{L}_{mod}\left(t\right)+\frac{Qo{L}_{mild}}{365}\sum_{t=0}^{j}{L}_{mild}\left(t\right)$$

Standardized QALYs across age groups were calculated by dividing cumulative QALYs by approximate size of the age group as reported by the 2020 California Department of Finance^22^. Standardized QALYs were also normalized by dividing cumulative QALYs by the total estimated long COVID cases by age group.

### COVID-19 case data

This analysis is limited to reported confirmed cases and is not a complete estimate of all COVID-19 infections since unreported or undetected infections can also go on to develop long COVID. While reported cases may still include some proportion of asymptomatic infections detected via routine surveillance screening, it is likely that reported cases are still underrepresenting possible asymptomatic infections. Between March 1, 2020, and December 31, 2022, there were 10,945,079 confirmed COVID-19 cases reported in California^23^. Time series of case counts were disaggregated by age (0–4, 5–17, 18–49, 50–64, 65+) and served as initial inputs into the infected individuals (*I*) compartment. Age and variant-specific hospitalization and mortality rates were calculated based on individual level data and incorporated into the model as fixed parameters to quantify incident (new QALYs per day) and cumulative QALYs. The three variant periods used were ancestral and Alpha (March 1, 2020–May 31, 2021), Delta (June 1-December 31, 2021), and Omicron (January 1–December 31, 2022). We limited the study period to March 1, 2020–December 31, 2022, to avoid lower ascertainment levels later in the pandemic due to the increased use of non-reportable at-home testing, although under-ascertainment occurred throughout the pandemic, particularly in the first half of 2020.

### Model parameterization

Based on prevalence estimates from a systematic review, we assumed 31% of infected individuals go on to experience long COVID, as this estimate is most closely aligned with our input data and study population (*p*_1_, Table 1)^24^. We used the CDC definition of long COVID and a time frame of 28 days to distinguish between acute and post-acute symptoms^2^. The average time individuals experience long COVID in the model depends on their level of symptom severity and ranges from 120 days (mild long COVID symptoms) to 247 days (severe long COVID symptoms after hospitalization). These durations can be calculated from the sum of the inverse of the rates in their model trajectory (Table 1). The proportion of cases associated with each long COVID severity level (mild, moderate, and severe) was derived from the National Institutes of Health (NIH) N3C database of individuals with confirmed SARS-CoV-2 infection and a long COVID diagnosis verified by ICD-10 code^25^. Disability weights for the three severity levels for ongoing symptoms were obtained from the Global Burden of Disease Study, which delineated these categories based on degree and type of interference with day-to-day activities (mild, moderate, severe) (Table 1)^9^. As our model separated long COVID status by severity instead of distinct symptom clusters (ongoing respiratory issues, cognitive problems, persistent fatigue), we averaged disability weight (QoL score) by severity level (Table 1). The QoL score for mild LC was the average for individuals with mild respiratory (0.02) and cognitive (0.07) problems. The QoL score for moderate LC was the average for individuals with moderate respiratory problems (0.23) and fatigue (0.22). The QoL score for severe LC was the average for severe respiratory (0.41) and cognitive (0.38) problems.

### Sensitivity analysis

**Fig. 2:**
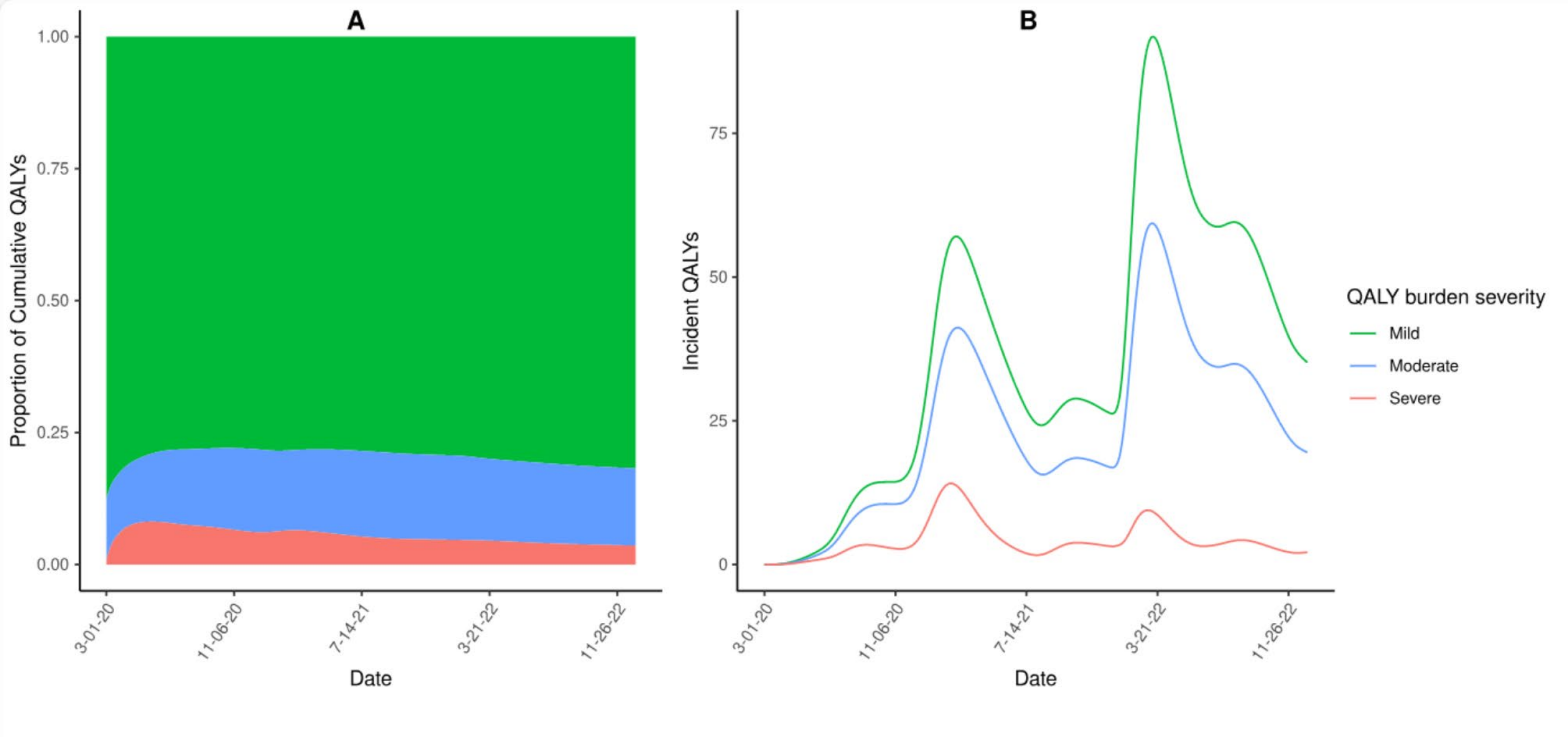
Estimated proportion of cumulative (**A**) and incident (**B**) QALYs lost due to long COVID among recovered SARS-CoV-2 cases in California, March 1st, 2020–December 31st, 2022, stratified by QALY burden severity.

Using Latin Hypercube Sampling (LHS) and partial rank correlation coefficients (PRCC), we performed a global sensitivity analysis to identify parameters that were most influential on model outcomes and gauge the robustness of results to epistemic uncertainty in parameter estimates^26,27^. LHS is a stratified Monte Carlo sampling method where each parameter’s range is divided into N intervals and each interval is sampled once, allowing for efficient sampling^28^. To maximize the depth of sampling, we set our N as 1000, which exceeds the minimum interval ratio of *N*$>\frac{4}{3}$*K* where K is equal to the number of parameters^29^. All parameter combinations were generated based on the distributions given in Table 1 using the lhs package in R^30^. Outcomes of interest were minimum and maximum QALYs (i.e. range) for severe, moderate, and mild long COVID, along with total QALYs. The sensitivity of outcomes to each model parameter was quantified with PRCC, a sampling-based sensitivity analysis method that measures monotonicity between two variables after removing the linear effects of all other parameters^28^. Parameters with PRCC values that are closer to 1 or −1 have a greater effect on model outcomes (Fig. 2).

## Results

**Fig. 3:**
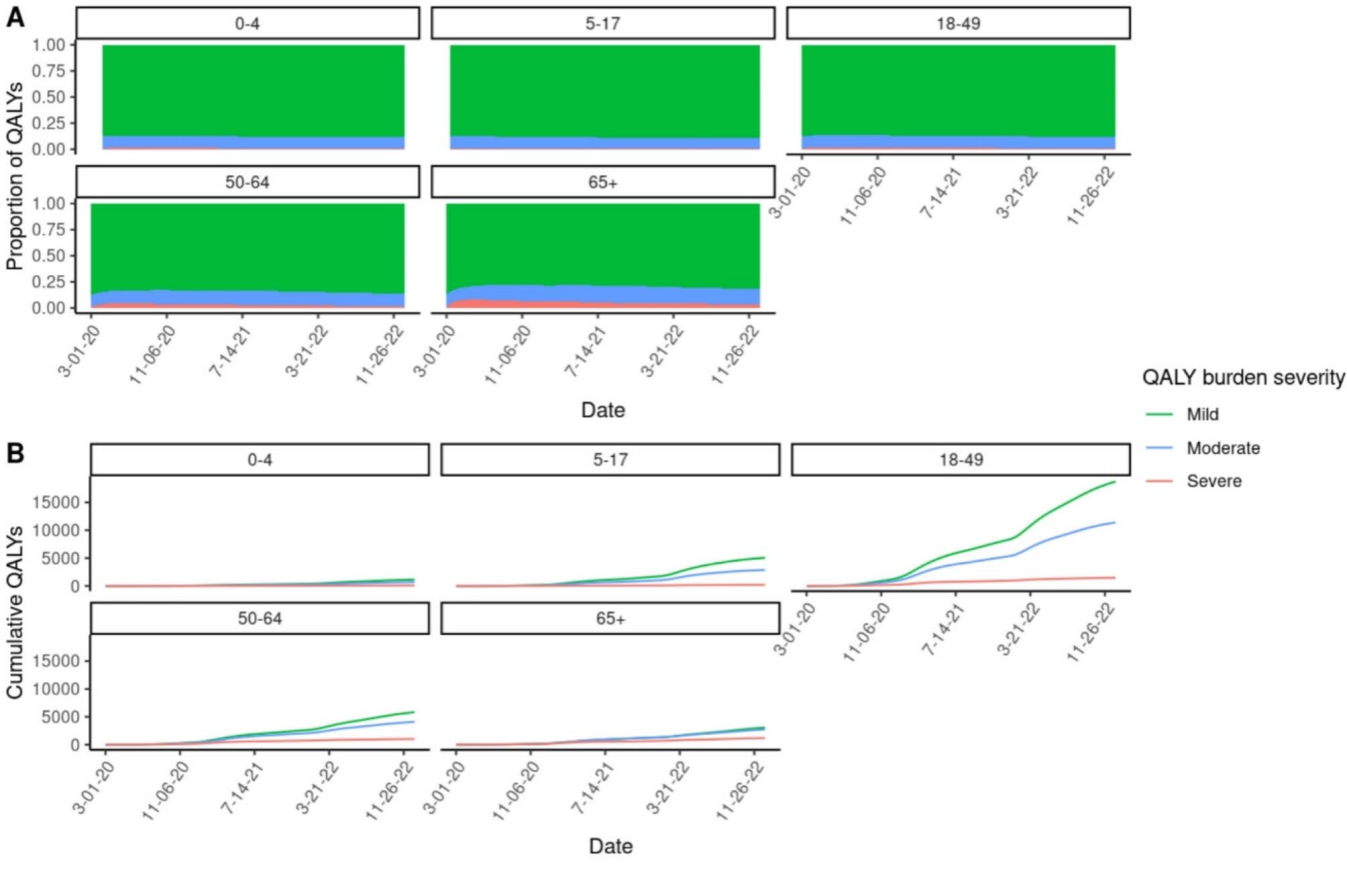
(**A**) Proportion of QALYs and (**B**) cumulative QALYs lost by level of long COVID symptom severity (mild, moderate, or severe) over time by recovering long COVID-19 cases by age group in California (March 1st, 2020-December 31st, 2022).

An estimated 59,514 QALYs (range: 10,372–180,257 QALYs from LHS), or 148.3 QALYs/100,000 population, were lost due to long COVID among confirmed COVID-19 cases in California between March 1st, 2020, and December 31st, 2022. On average, each COVID case contributed to 0.005 QALYs (59,514 total QALYs/10,945,079 cases) or 1.8 quality adjusted life days (QALDs). Among long COVID cases alone, the average loss per patient would be 59,514/3,300,137 = 0.0180 QALYs (6.6 QALDs). The overall and severity-specific burden of long COVID was not evenly distributed across age groups (Table 2). Children 0–4 lost 1,900 (1,900/59,514; 3.2%) cumulative QALYs or 81.7 QALYs/100,000 population, and children 5–7 lost 8,092 (8,092/59,514; 13.6%) cumulative QALYs or 120.8 QALYs/100,000 population. The majority of cumulative QALYs (31,592/59,514; 53.1%) and QALYs lost per 100,000 population (180.9) were in the 18–49-year-old age group. Adults 50–64 lost 10,945 (10,945/59,514; 18.4%) cumulative QALYs (148.1 QALYs/100,000 population), and adults 65 + lost 6,984 (6,984/59,514; 11.7%) cumulative QALYs (111.8 QALYs/100,000 population). When standardizing by the number of estimated long COVID cases, across all age groups there were 18.0 QALYs lost/1000 long COVID cases, which ranged from 16.8 QALYs lost/1000 long COVID cases among children 5–17 to a high of 22,0 QALYs lost/1000 long COVID cases among adults 65+. All age groups lost the majority of cumulative QALYs due to mild long COVID: 0–4 [89%], 5–17 [89%], 18–49 [88%], 50–64 [86%]), 65+ [82%] of cumulative QALYs from mild long COVID (Fig. 3).

The largest proportion of QALYs lost were due to mild long COVID, representing 87.5% of total burden (Fig. 2A). Moderate and severe disease accounted for 11.3% and 1.2% of cumulative QALYs lost, respectively. Incident QALYs lost spiked following the winter 2020–2021 COVID-19 surge in California^21^, and again in early 2022 with the Omicron wave (Fig. 2B). Despite the increases in incident QALYs lost, the proportion of QALYs that were lost due to severe and moderate symptoms peaked during the ancestral and Alpha period and declined during the Delta and Omicron variant periods (Fig. 2B).

**Table 2:** Estimated cumulative quality adjusted life years (QALYs), QALYs/100,000 population, and QALYs/1000 long COVID cases lost by confirmed COVID-19 cases in California disaggregated by age and long COVID severity level.

Age	Severe QALYs	Moderate QALYs	Mild QALYs	Total QALYs (QALYs per 100,000 population)	Total estimated long COVID cases* (QALYs per 1000 long COVID cases)
0–4	284	526	1,090	1,900 (81.7)	112,619 (16.9)
5–17	689	1,512	5,891	8,092 (120.8)	481,025 (16.8)
18–49	3,421	6,647	21,524	31,592 (180.9)	1,814,758 (17.4)
50–64	1,402	2,451	7,092	10,945 (148.1)	574,969 (19.0)
65+	1,366	1,775	3,843	6,984 (111.8)	316,766 (22.0)
All ages	7,162	12,911	39,441	59,514 (148.3)	3,300,137 (18.0)

*Note: numbers of estimated long COVID cases represent the first, most severe, severity level experienced by an individual, i.e., not the cumulative progression of an individual from severe to moderate to mild.

**Fig. 4:**
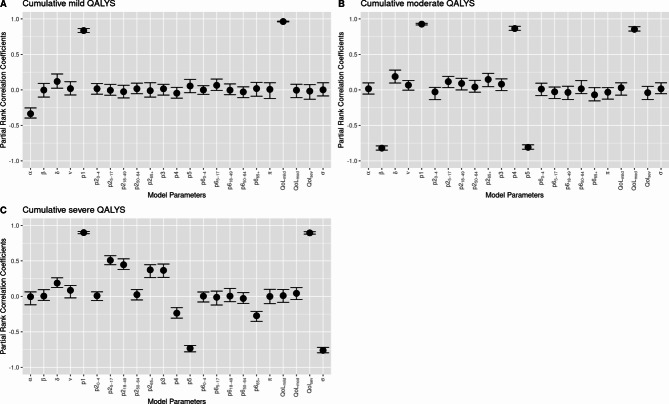
Sensitivity analysis results from LHS and PRCC of QALYs lost by recovered COVID-19 cases in California between March 1st, 2020-December 31st, 2022, due to cumulative mild (**A**), moderate (**B**), and severe (**C**) long COVID. Model parameters evaluated from left to right are (rate of recovery for mild long COVID symptoms), β (rate of symptom improvement from moderate to mild long COVID), $\sigma$, (rate at which an individual progresses from positive test result to recovery or long COVID onset), *p*_1_ (proportion infected that develop long COVID), *p*_2_ (percentage of individuals infected who become hospitalized during acute infection for ages 0–4, 5–17, 18–49, 50–64, and 65+), *p*_3_ (proportion severe long COVID), *p*_4_ (proportion severe long COVID), *p*_5_ (proportion severe long COVID), *p*_6_ (percentage of hospitalized individuals who die due to SARS-CoV-2 infection and related complications for ages 0–4, 5–17, 18–49, 50–64, and 65+), π (rate at which hospitalized individuals progress to death or develop severe long COVID symptoms), QoL_mild_ (disability weight for mild long COVID symptoms), QoL_moderate_ (disability weight for moderate long COVID symptoms), QoL_sev_ (disability weight for severe long COVID symptoms), and $\sigma$ (rate of symptom improvement from severe to moderate long COVID). For more detailed descriptions of parameters see Table 1.

The most influential parameters in the sensitivity analysis were the proportion of cases that develop long COVID (*p*_1_), severity proportion (*p*_3_, *p*_4_, *p*_5_), acute symptom duration (), time to long COVID ($\varDelta$), and QoL scores (Fig. 4). All outcomes were sensitive to and $\varDelta$, but less sensitive to symptom duration parameters permutations such as $\sigma$ or β. Mild QALYs were responsive to *p*_1_ and *p*_5_ but not to QoL score. QALYs associated with moderate long COVID were sensitive to the most variables: *p*_1_, *p*_4_, and moderate QoL score in addition to and $\varDelta$. QALYs associated with severe long COVID were sensitive to *p*_1_, *p*_3_ and only slightly to QoL_sev_.

## Discussion

We developed a framework to estimate the population burden of long COVID, incorporating severity level, age, and time-varying hospitalization and mortality rates that reflect the shifting SARS-CoV-2 variant landscape in California (Fig. 1). The proportion of QALYs lost due to severe manifestations of long COVID decreased through time for all age groups, which are largely due to increasing vaccination and population immunity (Fig. 2A). Since QoL values were assumed to be the same across age groups (Table 1), the differences in burden between age strata are the result of the combined effects of varying infection rates, hospitalization, and mortality rates. Compared to younger cases, adults 65 + experienced a higher proportion of estimated burden from moderate or severe symptoms, which reflects higher hospitalization rates among older patients (Fig. 3). Based on our assumption that severity of acute COVID infection (including hospitalization due to infection) is linked to severity of long COVID symptoms, higher hospitalization rates among certain age groups are highly informative to our understanding of burden severity that has not been captured by many existing studies. Together these results support the idea that long COVID burden should not be treated as a static value and that stratifying burden estimates by age and severity are important heterogeneities to capture.

Older adults should be prioritized for treatment and care of long COVID due to higher total healthcare usage, costs from acute infection, and severe long COVID in this group compared to younger individuals^31,32^. However, even mild long COVID symptoms can reduce productivity over time, due to reduced hours or scope of daily activities and/or work^7,33,34^. After accounting for population size, QALYs lost per 100,000 were still higher among 18–49 year olds compared to other age groups, suggesting that increased focus could be beneficial for this group. Additionally, younger individuals may experience long COVID (all severity levels) for a longer period due to their prospective lifespans, which would have further consequences for long term healthcare usage.

Compared to similar studies, we used higher disability weights, higher prevalence of symptoms, and longer symptom duration in our models, which may influence the accuracy of outcome estimation. The ranges of values used by other studies were more conservative, ranging from 0.129 to 0.318 for disability weights and 10–16.6% for prevalence estimates^11,35–37^. Empirical estimates of long COVID symptom duration were not available earlier in the pandemic, so other studies used a decay factor^11^, a duration of only 28 days^36,37^, or existing values for disability per case (QALYs lost per episode) instead^35^. The values used in this model are more current for long COVID, as many patients report the presence of ongoing symptoms at six months, one year, and two years post-infection^4,7,38^. Finally, we assumed that symptom duration was associated with severity level; i.e., individuals with severe symptoms have longer symptom duration because they experience a cascading decrease in symptom severity over time before recovery. Although this assumption is not commonly used in other modeling studies, individuals generally report fewer symptoms over time unless long COVID leads to a permanent disability^7,33,39^. Compared to some publications which estimate 11.1–75.2 QALDs, our estimate of 6.6 QALDs per long COVID patient is lower and reflects the simulation approach used which includes many people with mild long COVID. These other studies include persons who are more likely to report greater loss in quality of life, which might not reflect the distribution of long COVID at a broader population level^40,41^.

This model has several limitations, including inherent uncertainty surrounding the model structure and parameters. Several other factors that can impact long COVID-19 disease outcomes such as pre-existing conditions, sex, vaccination status, and history of prior infection were not explicitly included in the model due to lack of appropriate data to inform parameterization. The distribution of overall burden by age is partially due to unequal age group bands and testing demographics, however we did standardize our estimates per 100,000 population to account for this uneven distribution. We explored the inherent uncertainty surrounding model parameters in sensitivity analysis, as many were poorly measured or had limited data available to use in determining estimates and ranges. This variability was reflected in the range of expected total burden (10,372–180,257 QALYs lost), and care should be taken in interpreting the point values we reported in our main results. In addition to severity specific QoL score, duration of acute infection (), time between acute and long-term symptoms ($\varDelta$), proportion of individuals with long COVID (*p*_1_), and proportion of people with mild, moderate, and severe symptoms (*p*_3_, *p*_4_, *p*_5_) were some of the most influential parameters in the model (Fig. 4, eFigure 2). The proportion of individuals with long COVID was a key assumption, but accurate estimates of this value were difficult to obtain due to the use of contrasting definitions and criteria to define long COVID. We selected a point estimate (0.31 [0.09–0.53]) that best aligned with our case population, and we suggest that future studies also take the target population into account. Since the model does not explicitly account for individuals with asymptomatic or mild acute symptoms that did not receive a PCR test or used home antigen testing, using a higher long COVID prevalence may help account for unmeasured burden. Another assumption of our model was that the proportion of COVID-19 cases hospitalized among those who do and do not develop long COVID was equal. This is likely a conservative assumption for estimating long COVID burden; the true proportion of hospitalized cases who develop long COVID is likely higher than non-hospitalized cases^42^, and therefore our calculations are likely an underestimate of the true burden from hospitalized individuals. Estimates for symptom severity proportion (*p*_3_, *p*_4_, *p*_5_) were derived from ICD-10 codes in patient data^24^; individuals in this dataset may be more likely to be healthcare seeking and/or have comorbidities^43^. We chose to highlight the effects of differential symptom severity, and thus did not directly account for compounding or protective effects of repeated infection or vaccination in our model, which might alter the estimated proportion of individuals experience symptoms of differing severity levels over time. Additionally, disability weights for long COVID may differ by age, which we did not account for in this model due to lack of appropriate data. Although we did not evaluate differential burden by race and ethnicity, the odds of developing long COVID given confirmed acute SARS-CoV-2 infection are disproportionately high among people of color^44–46^, and this disparity needs to be addressed in future models to better serve marginalized populations.

Model accuracy over time is heavily dependent on data quality and reliability. The proportion of SARS-CoV-2 infections that are ascertained through healthcare and community testing has varied dramatically over the course of the pandemic and by age group^47^. In California, confirmed COVID cases are only from positive nucleic acid amplification tests (NAATs), which excludes infections identified by antigen tests; this model does not account for long COVID burden from unreported or probable infections, which means that burden due to mild long COVID is likely underestimated. In prior California serosurveys, up to 95% of COVID cases might be unreported to public health^48^. Additionally, even though current testing captures individuals who are more likely to experience moderate or severe symptoms, such as hospitalized individuals^9^, as testing decreases in response to changes in behavior and policy, official case counts may no longer be a reliable model input, even for capturing higher-risk symptomatic cases.

Mechanistic modeling allows us to explore parameter uncertainty, identify key parameters for future studies, and test assumptions about long COVID progression. Consideration of long COVID burden as a time-varying function of variant-specific infection and hospitalization allows for more nuanced estimates that can be used by public health practitioners and policymakers in decision making. Estimates of relative long COVID burden can allow public health to allocate or target resources, messaging surrounding testing or vaccination, and test-to-treat programs to persons and groups with the most severe or highest total proportion of burden^49^. Modeling can be a timely tool to fill in gaps in knowledge as there are currently few standardized surveillance methods for long COVID. Tracking disease burden with QALYs is also an approach that could also apply to other understudied chronic conditions. Other unmeasured, unobserved factors such as ascertainment and health disparities should be accounted for in future, stochastic models to estimate long COVID burden. Accurate measurement of parameters such as symptom duration and severity proportion and appropriate selection of parameters values that align with the target study population are also important for future model development. We present one potential theoretical framework to estimate long COVID burden in California that can be continuously modified and improved as more research on this condition is conducted.

## Conclusions

An age and variant-specific approach was used to estimate the burden of long COVID in California using modeling methods. Total and severity specific burden was unequal across age groups; the highest cumulative and per capita QALY burden was in adults 18–49, and adults 65+ faced more severe symptoms proportionally compared to younger age groups. Incorporating heterogeneities in considering long COVID gives a more realistic picture of burden to inform mitigation and resource allocation to affected individuals.

## Supplementary Information

Supplementary Material 1

## Data Availability

The data that support the findings of this study are available from California COVID-19 State Dashboard: https://covid19.ca.gov/state-dashboard/, but restrictions apply to the availability of these data, which were used under license in a summarized form for the current study, and so are not publicly available. To protect individual privacy, we can only share aggregated time series data. All R code and aggregated time series datasets used for analysis and figure generation are available in the public repository: https://github.com/sophiemzhu/longcovid. Cell sizes with <11 samples have been suppressed for privacy and are recoded to 0 in the publicly available code. The data are available from the corresponding author (S. Zhu) on reasonable request and with permission of CDPH.
