# Supplementary material for: Modeling the burden of long COVID in California with quality adjusted life-years (QALYS)
Source: Sci Rep. 2024 Sep 30;14:22663. doi: 10.1038/s41598-024-73160-x (PMC11443048; doi:10.1038/s41598-024-73160-x)
Supplement: Supplementary file 1 — Supplementary Material 1 [file 41598_2024_73160_MOESM1_ESM.docx]

**Online Supplemental Material**

**Modeling the burden of long COVID in California with quality adjusted life-years (QALYS)**

Sophie Zhu Ph.D.^1,2*^, Kalyani McCullough M.D.^1^, Jake M. Pry Ph.D.^1,3^, Seema Jain M.D.^1^, Lauren A. White Ph.D.^1^^, Tomás M. León Ph.D.^1^^


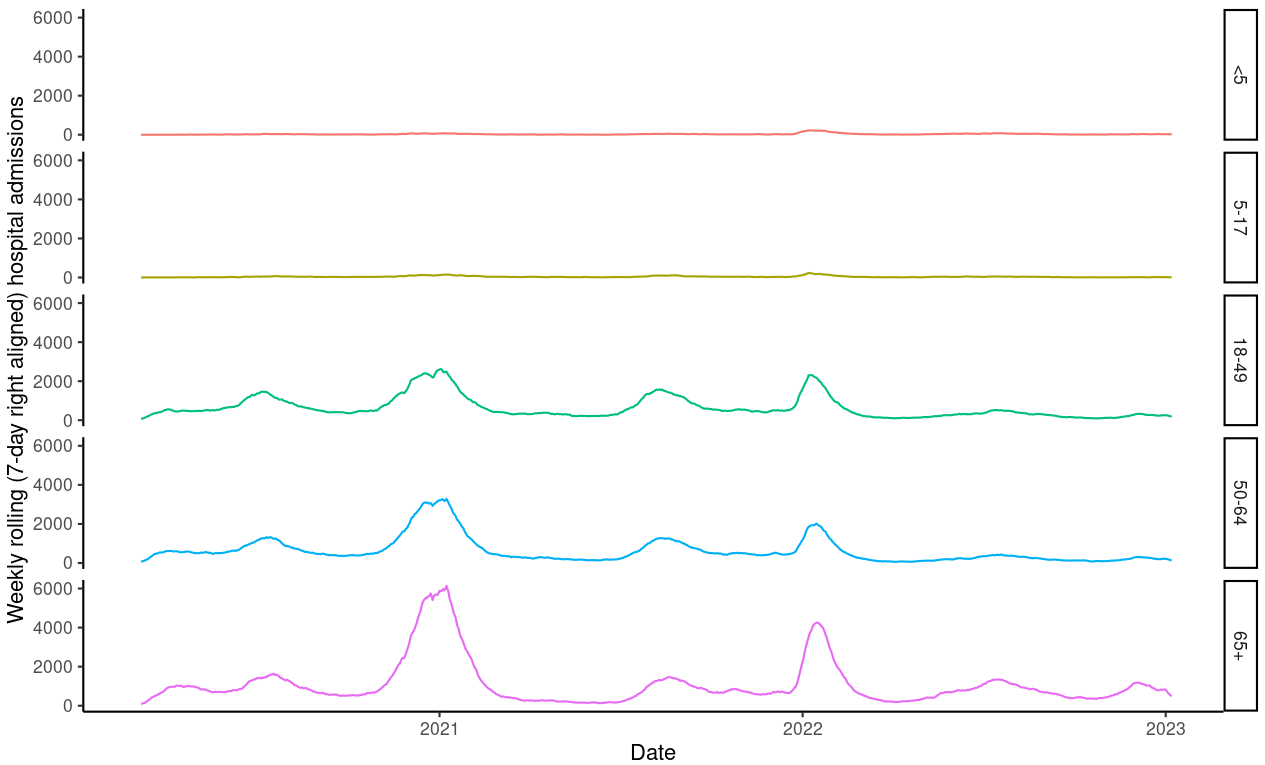


**eFigure 1.** Weekly hospital admissions (7-day rolling sum) due to SARS-CoV-2 infection between March 1st, 2020-December 31st, 2022 by age group.


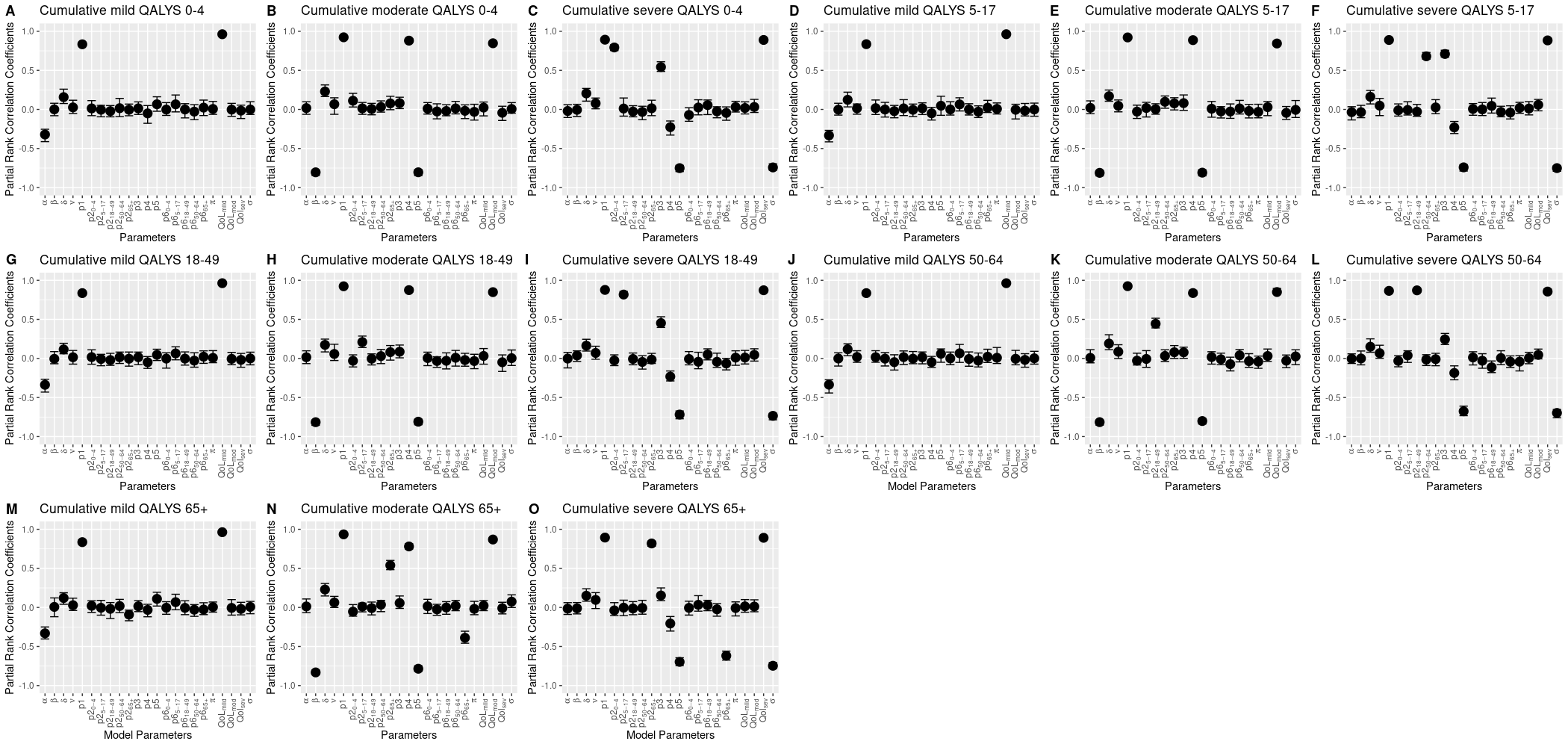


**eFigure 2**. Age specific sensitivity analysis results from LHS and PRCC of QALYs lost by recovering SARS-CoV-2 cases in California between March 1st, 2020-December 31st, 2022, due to mild, moderate, and severe long COVID.

**eTable 1**. Expanded description of parameter specification and selection for compartmental model of long COVID burden in California, 2020-2022

| Parameter | Description | Estimate | Details of parameter specification | Citation |
| --- | --- | --- | --- | --- |
| 𝑣 | Rate at which an individual progresses from positive test result to recovery or long COVID onset | 1/14 days^-1^ | We set a mean of 14 days to be the cutoff for recovery duration. In sensitivity analysis a range of 6.87 to 28 days was used to distinguish between acute and beginning of long COVID symptoms. | ^49^ |
| $\Delta$ | Rate at which an individual progresses from end of acute infection to beginning of long COVID symptoms | 1/14 days^-1^ | The cutoff we used for distinguishing acute symptoms and long COVID was 28 days. Since 14 days was used for 𝑣, the recovery rate, we assumed the amount of time to progress to long COVID was approximately equal. A range of 14-76 days was used in sensitivity analysis, which includes the cutoff of 90 days used in other definitions of long COVID. | ^2^ |
| π^b^ | Rate at which hospitalized individuals progress to death/recovery or develop severe long COVID symptoms | 1/5 days^-1^ | This estimate was based on COVID-19 case data internal to CDPH, where duration of illness before death was the difference between date of death and date first entered hospital. A range of 4-6 days was used in sensitivity analysis. | CDPH |
| $\sigma$^c,d^ | Rate of symptom improvement from severe to moderate long COVID | 1/40.66 days^-1^ | The rate of symptom improvement from severe to moderate long COVID symptoms was assumed to be a proportion of the estimated mean long COVID symptom duration among individuals who were hospitalized (9 months) from the referenced Hanson et al. 2022 study. We subtracted 28 days for the initial time from infection to development of long COVID symptoms already accounted for by parameters 𝑣 and $\Delta$, which gives the estimate of 270-28=242 days. We then assumed that persons with severe long COVID must move through all lower symptom severity categories before full recovery, and that the rate of recovery for mild long COVID symptoms (4 months) would be the same for persons previously hospitalized; the estimate was then 242-120 = 122 days. We assumed that individuals would spend approximately twice as long with moderate long COVID (81.33 or 2/3 of 122) compared to severe long COVID (40.66 or 1/3 of 122). Because there were few references to inform the estimate for proportion of time needed for each level of symptom improvement (severe to moderate vs moderate to mild) a range of 40.66-81.33 days was used in sensitivity analysis for both parameters. | [^9^](https://www.zotero.org/google-docs/?B4qJXK) |
| β^c,d^ | Rate of symptom improvement from moderate to mild long COVID | 1/81.33 days^-1^ | The rate of symptom improvement from moderate to mild long COVID symptoms was assumed to be a proportion of the estimated mean long COVID symptom duration among individuals who were hospitalized (9 months) from the referenced Hanson et al. 2022 study. We subtracted 28 days for the initial time from infection to development of long COVID symptoms already accounted for by parameters 𝑣 and $\Delta$, which gives the estimate of 270-28=242 days. We then assumed that persons with severe long COVID must move through all lower symptom severity categories before full recovery, and that the rate of recovery for mild long COVID symptoms (4 months) would be the same for persons previously hospitalized; the estimate was then 242-120 = 122 days. We assumed that individuals would spend approximately twice as long with moderate long COVID (81.33 or 2/3 of 122) compared to severe long COVID (40.66 or 1/3 of 122). Because there were few references to inform the estimate for proportion of time needed for each level of symptom improvement (severe to moderate vs moderate to mild) a range of 40.66-81.33 days was used in sensitivity analysis for both parameters. | [^9^](https://www.zotero.org/google-docs/?EozdYr) |
| 𝛼^d,e^ | Rate of recovery for mild long COVID symptoms | 1/92 days^-1^ | The rate of recovery for mild long COVID symptoms was assumed to be the estimated mean long COVID symptom duration among individuals who were not hospitalized (4 months) from the referenced Hanson et al. 2022 study. We subtracted 28 days for the initial time from infection to development of long COVID symptoms already accounted for by parameters 𝑣 and $\Delta$, which gives the estimate of 120-28=92 days. A range of 80-110 days was used in sensitivity analysis based on the confidence intervals described by Hanson et al., again accounting for the 28 days already included as prior rates in this model. | [^9^](https://www.zotero.org/google-docs/?GJDkEt) |
| *p*_1_^f^ | Proportion infected that develop long COVID | 0.31 | We used a higher estimate for proportion of infected individuals that develop long COVID to help account for underreporting of cases with asymptomatic and mild disease that may not be captured with NAAT tests in California. A triangle distribution was used because it was assumed that the population prevalence of long COVID in the California surveillance data would be lower and closer to those found in the CDC’s Household Pulse survey (<15%) rather than the upper bound of 53% reported in the cited meta-analysis. This upper bound represents populations that are more likely to be hospitalized, which is not as reflective of the population captured by statewide surveillance. | [^6^](https://www.zotero.org/google-docs/?SwJVN9) |
| *p*_2_^b^ | Percentage of individuals infected who become hospitalized during acute infection | (Age: Alpha, Delta, Omicron)^g^  0-4: 0.016, 0.015, 0.011  5-17: 0.007, 0.005, 0.002  18-49: 0.024, 0.021, 0.007  50-64: 0.078, 0.065, 0.019  65+: 0.222, 0.167, 0.083 | These estimates were based on COVID-19 case data internal to CDPH. Percentages were calculated as number of cases hospitalized during acute infection over total cases, stratified for each of the COVID-19 variant periods included in the model. | CDPH (eFigure1) |
| *p*_3_ | Proportion severe LC | 0.005 | The proportion of persons with severe long COVID was estimated from the National COVID Cohort Collaborative (NC3) dataset through December 31, 2022. As the proportion of enrollees with severe/moderate/mild long COVID may be highly dependent on the population enrolled, a range of -50% to +50% were used as conservative upper bounds for sensitivity analysis. | NIH data |
| *p*_4_ | Proportion moderate LC | 0.117 | The proportion of persons with severe long COVID was estimated from the National COVID Cohort Collaborative (NC3) dataset through December 31, 2022. As the proportion of enrollees with severe/moderate/mild long COVID may be highly dependent on the population enrolled, a range of -50% to +50% were used as conservative upper bounds for sensitivity analysis. | NIH data |
| *p*_5_ | Proportion mild LC | 0.878 | The proportion of persons with severe long COVID was estimated from the National COVID Cohort Collaborative (NC3) dataset through December 31, 2022. As the proportion of enrollees with severe/moderate/mild long COVID may be highly dependent on the population enrolled, a range of -50% to +50% were used as conservative upper bounds for sensitivity analysis. | NIH data |
| *p*_6_^b^ | Percentage of hospitalized individuals who die due to SARS-CoV-2 infection and related complications | (Age: Alpha, Delta, Omicron)^g^  0-4: 0.006, 0.009, 0.005  5-17: 0.008, 0.009, 0.011  18-49: 0.085, 0.107, 0.051  50-64: 0.231, 0.237, 0.159  65+: 0.541, 0.390, 0.289 | These estimates were based on COVID-19 case data internal to CDPH. Percentages were calculated as number of hospitalized cases who died over total cases hospitalized, stratified for each of the COVID-19 variant periods included in the model. | CDPH |
| QoL_mild_ | Disability weight of individuals with mild respiratory and cognitive problems (cough and shortness of breath after physical exertion, difficulty in concentrating and remembering recent events) | 0.045 (0.005, 0.10) | The QoL score for mild LC was the average for individuals with mild respiratory (0.02) and cognitive (0.07) problems. The lowest and highest bounds for both categories were used in sensitivity analysis. | [^9^](https://www.zotero.org/google-docs/?lR6Ppe) |
| QoL_mod_ | Disability weight of individuals with moderate chronic respiratory and/or persistent fatigue with bodily pain or mood swings (cough and shortness of breath after light physical activity, always feel fatigue and may have depression) | 0.225 (0.15-0.31) | The QoL score for moderate LC was the average for individuals with moderate respiratory problems (0.23) and fatigue (0.22). The lowest and highest bounds for both categories were used in sensitivity analysis. | [^9^](https://www.zotero.org/google-docs/?1OMTYw) |
| QoL_sev_ | Disability weight of individuals with severe chronic respiratory problems and cognitive problems (cough and shortness of breath all the time, disorientation, memory problems, and confusion to where one needs help with some daily activities) | 0.395 (0.20-0.59) | The QoL score for severe LC was the average for severe respiratory (0.41) and cognitive (0.38) problems. The lowest and highest bounds for both categories were used in sensitivity analysis. | [^9^](https://www.zotero.org/google-docs/?mP9EBB) |
